# Prevalence and correlates of onychophagia among Egyptian medical students: a cross-sectional study

**DOI:** 10.1007/s44192-025-00327-x

**Published:** 2025-12-21

**Authors:** Asmaa Mohammad Ahmad Mohammad, Rabab Ahmed Abd El-Hai Hammad, Noha M. Elghazally

**Affiliations:** Department of Public Health and Community Medicine, Faculty of Medicine, Tanta University, Tanta City, Egypt

**Keywords:** Onychophagia, Medical students, Prevalence, Behavioural

## Abstract

**Background:**

Onychophagia, or chronic nail biting, is a common body-focused repetitive behaviour often associated with psychological stress, anxiety, and perfectionism. Medical students may be at increased risk due to sustained mental and emotional pressures.

**Objectives:**

To assess the prevalence of onychophagia, identify associated risk factors, and examine the relationship between perceived stress levels and the frequency or severity of nail biting among medical students at Tanta University in Egypt.

**Methods:**

A cross-sectional study was conducted among 330 undergraduate students using a structured self-administered questionnaire comprising sociodemographic data, onychophagia-related items, and the Perceived Stress Scale (PSS).

**Results:**

57.3% of the students were actively practicing onychophagia, and it was reported more often among males (63.5%) than females (36.5%), but the difference was not statistically significant (*p* = 0.134). Among participants with onychophagia (*n* = 189), 18 (9.5%) were smokers, 63 (33.3%) reported a positive family history, and 30 (15.9%) reported fear of public spaces. These factors were significantly associated with nail-biting behaviour (*p* < 0.01 for all). No significant difference in Perceived Stress Scale scores was observed between students with and without onychophagia, and stress severity was not associated with the frequency or duration of nail-biting episodes.

**Conclusion:**

Onychophagia was common among medical students and was significantly associated with smoking, positive family history, and fear of public spaces. Behavioural interventions are recommended among university students.

## Introduction

Onychophagia, or habitual nail biting, is chronic nail problem that typically first appears in childhood or early adulthood [1, 2]. Most nail biters eventually stop eating their nails [3]. Nevertheless,, in certain individuals, it might continue into adulthood [4]. Onychophagia, which ranges in severity from mild to severe, is an issue that is often overlooked in routine clinical treatment [5, 6]. 

Oral habits prevalence has increased worldwide [7–9]. Nail biting is estimated to affect approximately 20% to 30% of the general population [10], with higher rates observed among children. For instance, one study conducted in Egypt reported a prevalence of 41.07% among schoolchildren aged 6 to 12 years [11]. Populations exposed to elevated stress levels, such as university students, also demonstrate a higher incidence of this behaviour [1], with medical students exhibiting the highest rates [3]. A study from Poland found that the prevalence of onychophagia among university students aged 21 to 26 years reached 46.9%.^(3)^ However, evidence regarding sex-associated differences in prevalence remains inconclusive, with some studies indicating a male predominance, others suggesting higher rates among females, and several reporting no significant difference. ^( 3)^

The aetiology of onychophagia remains unclear [12]. However, familial patterns appear to contribute to its development, suggesting a possible genetic or environmental component [13]. Previous studies have reported that up to 36.8% of individuals with nail-biting habits have family members who exhibit the same behaviour [13]. Additionally, some research indicate that nail biting may be triggered by either over-stimulation, such as stress or excitement, or under-stimulation as boredom or inactivity [5, 14]. 

Onychophagia has been associated with various coexisting psychiatric conditions [15], including OCD, anxiety disorders, and tic disorders [16]. While traditionally linked to obsessive-compulsive behaviour, recent studies suggest it may be more accurately classified as a tic disorder [16–18] The ICD-10 categorizes nail biting under behavioural and emotional disorders of childhood and adolescence (F98.8) [19], whereas the DSM-5 identifies it as a body-focused repetitive behaviour [20]. Diagnostic criteria for such behaviours remain poorly defined, though persistent, unsuccessful attempts to stop the habit despite social or emotional consequences are a key feature [20]. 

Onychophagia can lead to a variety of adverse health outcomes [21]. These include dental issues, such as malocclusion and enamel damage, as well as infections of the oral cavity and gastrointestinal disturbances caused by the ingestion of nail fragments [21]. In more severe cases, excessive nail biting may result in splinter haemorrhages, scarring of the nail folds, paronychia, and even irreversible deformation of the nail beds [21]. Beyond its physical complications, onychophagia has been linked to a reduced quality of life, elevated stress levels, psychiatric comorbidities, social difficulties, and overall poorer physical health [3]. 

Although onychophagia is recognized as a medical condition and is associated with adverse health outcomes, there is limited research on its prevalence among both youth and adult populations [22]. Medical students, as future healthcare providers, represent a particularly relevant population in which mental health and self-care behaviours are of critical importance. Therefore, the aim of this study was to assess the prevalence of onychophagia, to identify sociodemographic and behavioural risk factors associated with nail biting, and to test whether higher perceived stress levels are linked to increased frequency and greater severity of onychophagia.

## Study design, duration, and setting

A cross-sectional study was conducted between November 2024 and February 2025 at the Faculty of Medicine, Tanta University, Egypt.

### Study population

The inclusion criteria comprised medical students from the first to the fifth academic year, representing approximately one to five years since admission to the university. Including students across all academic levels allowed for broader representation of both preclinical and clinical stages. Eligible participants were those who were currently engaging in onychophagia or had previously engaged in the behaviour during the study period and expressed a willingness to participate. Prior to participation, students received a brief explanation of the study objectives and were invited to voluntarily complete the study questionnaire.

## Sampling technique and sample size

Participants were selected using a non-randomized convenience sampling technique, based on accessibility and willingness to participate rather than random selection. As participation was voluntary and not all medical students had an equal probability of inclusion. Considering a 27.8% prevalence of onychophagia among university students [23], with a precision of 5% and a 95% confidence interval, the minimum proper sample size of participants was determined to be 295. The overall sample size included 330 students after considering the percentage of the drop out cases. Sample size determined utilizing EPi Info. The participants were informed about the study’s objectives and benefits. The response rate was 98%.

## Study measures

A self-administered questionnaire adapted from two validated questionnaires [3, 23], was utilized in this study. That combined instrument showed validation and reliability, with a Cronbach’s Alpha of 0.87. The questionnaire consisted of four sections. The first section collected information on sociodemographic characteristics, including age, gender, academic year, and family income. The second section focused on aspects of the participants’ lifestyle and health status (smoking, chronic diseases, personal and family history of mental disorders or nail-biting).

The third part included onychophagia-related items which were adapted from two previously validated questionnaires [3, 23]. These items covered the frequency, duration, triggers, associated feelings, and consequences of nail-biting. The severity of onychophagia was evaluated across three dimensions: frequency, daily episodes, and duration of each episode. Based on self-reported behaviours, frequency was categorized as mild when nail biting occurred several times per month, moderate when it occurred several times per week, and severe when it was practiced daily. The frequency of episodes per day was classified as mild for < 5 episodes, moderate for 5–20 episodes, and severe for more than 20 episodes per day. Regarding the duration of each episode, the habit was considered mild when lasting 1–10 min per day, moderate when lasting 11–20 min per day, and severe when exceeding 20 min per day. To ensure content validity, the items were reviewed by two experts in psychiatry and public health, who confirmed that the wording was appropriate and culturally relevant for Egyptian medical students. A pilot test was conducted on a small group of students (not included in the final analysis) to check for clarity and comprehensibility. Feedback from that pilot was used to refine the language of certain items. The onychophagia-related items demonstrated good internal consistency in the study sample (Cronbach’s α = 0.78).

The fourth section included a modified Perceived Stress Scale (PSS) were used to assess participants’ stress levels over one month before the study [24]. Higher scores indicate higher levels of perceived stress. The scale ranged from “never = zero “to “always = four”, taking into account that there are two reversed score items which means “always = zero” and “never = 4” [25]. After taking the opinions of two experts, we used only eight items out of ten items of the scale with a total score of 32. The PSS used in its validated form in English Two experts in psychiatry and public health confirmed that the wording was appropriate and culturally relevant for Egyptian medical students. Additionally, the modified 8-item version (excluding items 5 and 8 for clarity) was pretested on a pilot group of students to ensure understanding. The internal consistency (Cronbach’s alpha) of the modified scale in the study sample was 0.81 indicating acceptable reliability. The results were classified as: low stress level (0:10), moderate (11:21) and high (22:32) [26]. 

### Statistical analysis

Analysis was performed utilizing the Statistical Package for Social Sciences (SPSS) (version 22.0, IBM, Armonk, NY). A descriptive analysis of all study variables was conducted including frequencies (n, %), means, standard deviations (SD), medians and interquartile ranges (IQR). The chi-square test was used to compare between categorical outcomes. Other tests of association were used when Chi-square test was not appropriate like Fisher’s Exact test (FE) and Monte-Carlo Exact test (MCET). Risk estimation was done using Odd’s Ratio (OR) depending on the 95% Confidence Interval (CI). Diagnostic Odd’s Ratio was detected through Receiver Operating Characteristic curve (ROC curve). Receiver Operating Characteristic (ROC) curve analysis was used to evaluate the diagnostic performance of the main predictor variables. The continuous outcomes were compared using an independent t-test and Mann-Whitney U test according to the results of the normality tests. Tests of correlation were used like Eta and Phi to detect and describe the correlations between the different types of categorical data. Binary logistic regression analysis was used to estimate the amounts of correlations between the dependent qualitative dichotomous variable and the different tested independent variables. Level of significance was established at *p* < 0.05.

Controlling of the confounders was done as follows: Regarding the smoking habit “after adjusted OR by using Cochran’s and Mantel-Haenszel technique”; OR after nullifying the effect of sex = 4.471, 95% CI = 1.285–15.554. OR after nullifying the effect of nail infections = 4.832, 95% CI = 1.401–16.668. OR after nullifying the effect of the panic disorder = 4.853, 95% CI = 1.382–17.041. Regarding agoraphobia “after adjusted OR by using Cochran’s and Mantel-Haenszel technique”; OR after nullifying the effect of sex = 7.056, 95% CI = 2.345–21.233. OR after nullifying the effect of nail infections = 6.432, 95% CI = 2.215–18.683. OR after nullifying the effect of the specific phobia = 6.152, 95% CI = 2.160-17.524. The family history of onychophagia “after adjusted OR by using Cochran’s and Mantel-Haenszel technique”; OR after nullifying the effect of the specific phobia = 4.930, 95% CI = 2.582–9.413. OR after nullifying the effect of the obsessive-compulsive disorder = 4.868, 95% CI = 2.555–9.274.

The missing data was handled as follows: We had very few missing data (less than 10% of the total participants) in some qualitative and quantitative variables of the third section of the questionnaire. Regarding the qualitative variables, using the Statistical Package for Social Sciences (SPSS) (version 22.0, IBM, Armonk, NY). By the help of the order of “transform” and “replace missing values” of such qualitative variables using {the median of nearby points} and after detection of the span of nearby points, the missing data were replaced. Regarding the quantitative variables, using the Statistical Package for Social Sciences (SPSS) (version 22.0, IBM, Armonk, NY). By the help of the order of “transform” and “replace missing values” of such quantitative variables using {the mean of nearby points} and after detection of the span of nearby points, the missing data were replaced.

## Results

Table 1 shows the distribution of the study participants without and with onychophagia by characteristics. Among 330 undergraduate medical students who participated in the study, 189 students (57.3%) reported current onychophagia. The mean age of students was 20.86 ± 1.453 and 21.20 ± 1.465 for students with and without onychophagia, with a statistically significant difference between them (*p* = 0.036). Among the total sample, 198 students (60.0%) were males and 132 (40.0%) were females. Within the onychophagia group, 120 of 189 (63.5%) were males and 69 of 189 (36.5%) were females, compared with 78 of 141 (55.3%) and 63 of 141 (44.7%) in the non-onychophagia group, respectively. The difference was not statistically significant (χ^2^ = 2.248, *p* = 0.134). Regarding the academic years, 39.7% and 52.5% of participants with and without onychophagia were in 5th academic year, respectively, with a statistically significant difference (*p* = 0.026). Family income did not differ significantly between groups (χ^2^ = 1.149, *p* = 0.284); the majority of both nail-biters and non-biters reported medium income (149/189 = 78.8% vs. 113/141 = 80.1%).

**Table 1:** Distribution of the study participants without and with onychophagia by characteristics

Characteristic		Onychophagia		*P-value*
		N (%)		
Socio-demographic characteristics		Without 141 (42.7)	With 189 (57.3)	
Age (years) Mean ± SD		21.20 ± 1.465	20.86 ± 1.453	0.036*
Sex	Male	78 (55.3)	120 (63.5)	0.134
	Female	63 (44.7)	69 (36.5)	
Academic year	1st year	15 (10.6)	28 (14.8)	0.026*
	2nd year	18 (12.8)	32 (16.9)	
	3rd year	14 (9.9)	25 (13.2)	
	4th year	20 (14.2)	29 (15.3)	
	5th year	74 (52.5)	75 (39.7)	
Family income	High	20 (14.2)	33 (17.5)	0.284
	Medium	113 (80.1)	149 (78.8)	
	Low	8 (5.7)	7 (3.7)	

Table 2 shows the distribution of the study participants with/without onychophagia regarding their life-style and health status. Smoking was reported by 18 of 189 (9.5%) students with onychophagia and 3 of 141 (2.1%) students without onychophagia. The difference was statistically significant (χ^2^ = 7.414, df = 1, *p* = 0.006). Smokers were almost five times more likely to exhibit onychophagia (OR = 4.842, 95% CI 1.398–16.777). Fear of public spaces was reported by 30 of 189 (15.9%) students with onychophagia versus 4 of 141 (2.8%) students without onychophagia (χ^2^ = 14.850, df = 1, *p* < 0.001; OR = 6.462, 95% CI 2.221–18.802). Regarding the family history, a positive family history of onychophagia was found in 63 of 189 (33.3%) participants with onychophagia compared to 13 of 141 (9.2%) among those without (χ^2^ = 26.489, *p* < 0.001; OR = 4.923, 95% CI 2.581–9.390).

**Table 2:** Distribution of the study participants without and with onychophagia by characteristics

Characteristic		Onychophagia		*P-value*	*OR*	*95% CI*
		N (%)				
Lifestyle & health status		Without	With			
		141 (42.7)	189 (57.3)			
Smoking habit	No	138 (97.9)	171 (90.5)	0.006*	4.842	1.398–16.777
	Yes	3 (2.1)	18 (9.5)			
History of Chronic diseases	No	122 (86.5)	162 (85.7)	0.833	1.070	0.569–2.014
	Yes	19 (13.5)	27 (14.3)			
Feeling anxious most of the time	No	107 (75.9)	134 (70.9)	0.313	1.292	0.786–2.124
	Yes	34 (24.1)	55 (29.1)			
History of panic episodes	No	127 (90.1)	157 (83.1)	0.069	1.849	0.946–3.614
	Yes	14 (9.9)	32 (16.9)			
Fear of public spaces	No	137 (97.2)	159 (84.1)	0.001*	6.462	2.221–18.802
	Yes	4 (2.8)	30 (15.9)			
Social anxiety	No	118 (83.7)	143 (75.7)	0.076	1.650	0.946–2.880
	Yes	23 (16.3)	46 (24.3)			
A strong fear of something specific	No	129 (91.5)	170 (89.9)	0.635	1.201	0.563–2.564
	Yes	12 (8.5)	19 (10.1)			
Nail infections	No	136 (42.5)	184 (57.5)	0.749	0.739	0.210–2.604
	Yes	5 (50)	5 (50)			
Family history of onychophagia	No	128 (90.8)	126 (66.7)	0.001*	4.923	2.581–9.390
	Yes	13 (9.2)	63 (33.3)			
Family history of mental disorders	No	127 (90.1)	173 (91.5)	0.647	.839	0.395–1.781
	Yes	14 (9.9)	16 (8.5)			

**Fig. 1:**
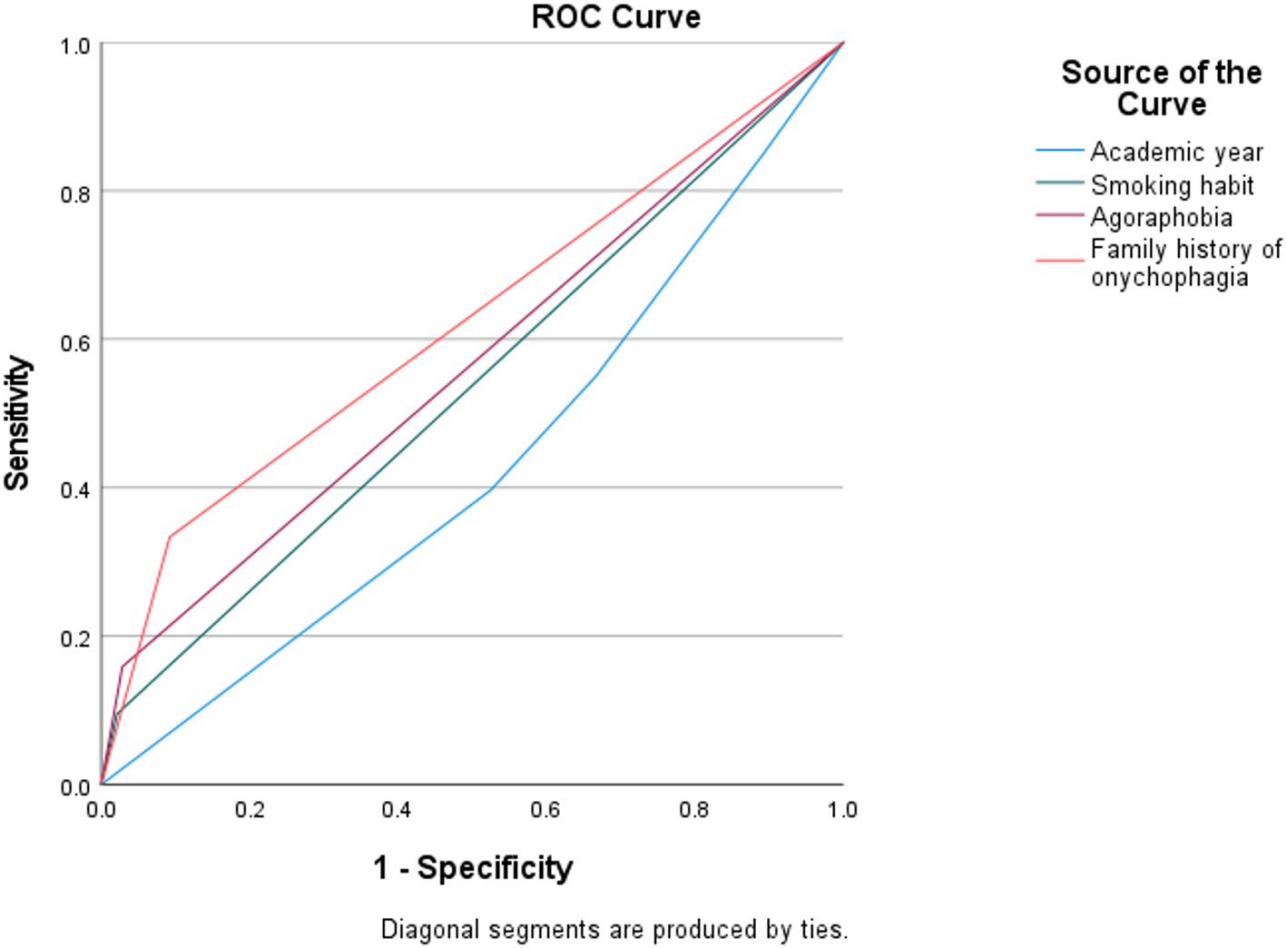
Receiver operating characteristic (ROC curve) for the different possible risk factors for practicing onychophagia

Figure 1 illustrates that there were significant weak positive correlations between participants’ family history of onychophagia, having fear of public spaces, smoking habit of the study participants and their practice of onychophagia (AUC = 0.621, 0.565 and 0.537, respectively). The values of correlation coefficients according to Phi test were 0.283, 0.212 and 0.150, respectively. The resulting r^2^ values (r^2^ = 0.080, 0.045 and 0.022). were then used to estimate the percentage of variance in onychophagia explained by each factor. Effect sizes were interpreted as small (*r* ≈ 0.1), moderate (*r* ≈ 0.3), and large (*r* ≥ 0.5), according to Cohen’s conventions. The overall effect size of those three factors on practicing onychophagia was 19.2% (Nagelkerke R Square = 0.192). Additionally, a small effect size (1.7%) of the educational level presented by the academic year of the study participants on their practice of onychophagia was detected (AUC = 0.429).

Table 3 shows the nail-biting habit according to the gender of the study participants, revealing a statistically significant difference recorded between males and females regarding eating finger nails; 25 of 69 (36.2%) of females reported never eating their fingernails while practicing onychophagia compared to 27 of 120 (22.5%) of males (χ^2^ = 5.644, *p* = 0.018). Regarding localization of nail-biting, severity of the habit and practicing it to achieve perfect look, no statistically significant differences were detected. Overall, the frequency and duration of nail-biting were nearly equal among males and females, 101 of 120 (84.2%) of males and 60 of 69 (87%) of females reporting mild to moderate frequency of onychophagia, 88.3% of males versus 95.7% of females reporting mild to moderate frequency of episodes per day and 110 of 120 (91.7%) of males versus 65 of 69 (94.2%) of females reporting mild to moderate durations of episodes per day (*p* > 0.05).

**Table 3:** Description of the habit according to sex of the study participants

Description of the onychophagia habit		Sex (n = 189)				*P-value*
		Male		Female		
		n	%	n	%	
		120	63.5	69	36.5	
Localization of nail biting	One hand	27	22.5	22	31.9	0.238
	Both hands	62	51.7	35	50.7	
	Selected fingernails	31	25.8	12	17.4	
Eating fingernails	Never	27	22.5	25	36.2	0.018*
	Almost never	35	29.2	23	33.3	
	Sometimes	38	31.7	14	20.3	
	Often	11	9.2	4	5.8	
	Always	9	7.5	3	4.3	
To achieve perfect look	Never	27	22.5	8	11.6	0.676
	Almost never	17	14.2	16	23.2	
	Sometimes	43	35.8	28	40.6	
	Often	23	19.2	13	18.8	
	Always	10	8.3	4	5.8	
Last nail-biting episode	> 6 months	34	28.3	11	15.9	0.079
	The last 6 months	22	18.3	13	18.8	
	The last month	26	21.7	18	26.1	
	The last week	16	13.3	9	13	
	Yesterday	5	4.2	4	5.8	
	Today	17	14.2	14	20.3	
Stopping for one month or longer	No	38	31.7	19	27.5	0.096
	Yes	46	38.3	37	53.6	
	Do not know	36	30.0	13	18.8	
Controlling the habit	Long term	49	40.8	23	33.3	0.308
	Short term	71	59.2	46	66.7	
Severity of the habit:						
Frequency of onychophagia	Mild to moderate	101	84.2	60	87.0	0.603
	Severe	19	15.8	9	13.0	
Frequency of episodes / day	Mild to moderate	106	88.3	66	95.7	0.090
	Severe	14	11.7	3	4.3	
Duration of episodes / day	Mild to moderate	110	91.7	65	94.2	0.522
	Severe	10	8.3	4	5.8	

Table 4 illustrates the PSS among participants with and without onychophagia, there was no statistically significant difference between the two groups regarding the total PSS scores (*p* = 0.113), as the majority of participants in both groups reported moderate stress levels. Students with onychophagia had the same median (PSS) score (15) as that of those without onychophagia (15). Taking in consideration the inter-quartile range of the (PSS) score of the first group which was (13–18) and that of the second group which was (12–17), the difference was not statistically significant (Mann-Whitney U test = 12099.000, *p* = 0.152), and the effect size was small (η^2^ = 0.013),

**Table 4:** Perceived Stress Scale (PSS) among study participants without and with onychophagia

Perceived stress scale (PSS)		Onychophagia		*P-value*
		Without n(%)	With n(%)	
Being upset because of something that happened unexpectedly	Never/Almost never	141(100.0)	189 (100.0)	–
	Sometimes	0 (0.0)	0 (0.0)	
	Fairly often/Very often	0 (0.0)	0 (0.0)	
Feeling being unable to control the important things in the life	Never/Almost never	29 (20.6)	54 (28.6)	0.133
	Sometimes	50 (35.5)	70 (37.0)	
	Fairly often/Very often	62 (44.0)	65 (34.4)	
Feeling nervous and stressed	Never/Almost never	18 (12.8)	39 (20.6)	0.173
	Sometimes	53 (37.6)	64 (33.9)	
	Fairly often/Very often	79 (49.6)	86 (45.5)	
Feeling confident about the ability to handle the personal problems	Never/Almost never	36 (25.5)	62 (32.8)	0.336
	Sometimes	57 (40.4)	72 (38.1)	
	Fairly often/Very often	48 (34.0)	55 (29.1)	
Finding that he/she could not cope with all the things that he/she had to do	Never/Almost never	33 (23.4)	52 (27.5)	0.679
	Sometimes	59 (41.8)	77 (40.7)	
	Fairly often/Very often	49 (34.8)	60 (31.7)	
Being able to control irritations in the life	Never/Almost never	37 (26.2)	46 (24.3)	0.651
	Sometimes	68 (48.2)	86 (45.5)	
	Fairly often/Very often	36 (25.5)	57 (30.2)	
Being angered because of things that were outside of his/her control	Never/Almost never	20 (14.2)	54 (28.6)	0.007*
	Sometimes	71 (50.4)	74 (39.2)	
	Fairly often/Very often	50 (35.5)	61 (32.3)	
Feeling difficulties were piling up so high that he/she could not overcome them	Never/Almost never	31 (22.0)	56 (29.6)	0.293
	Sometimes	68 (48.2)	81 (42.9)	
	Fairly often/Very often	42 (29.8)	52 (27.5)	
Total score	Low stress level	24 (17.0)	31 (16.4)	0.113
	Moderate stress level	103 (73.0)	150 (79.4)	
	High stress level	14 (9.9)	8 (4.2%)	
Medians of Total PSS scores (Inter-Quartile Range)		15 (13–18)	15 (12–17)	Mann–Whitney U test = 12,099.000 *p* = 0.152

η^2^ = 0.013 (Eta squared; effect size). *p* < 0.05 considered statistically significant. According to Cohen (1988), η^2^ = 0.01 indicates a small effect, 0.06 a medium effect, and 0.14 a large effect

Table 5 illustrates that among medical students with onychophagia, the severity, frequency, and duration of onychophagia were not significantly associated with perceived stress levels (Monte Carlo Exact Test, *p* > 0.05).

**Table 5:** Relation between stress and severity of onychophagia

Severity of onychophagia			Degree of stress						P-value*
			Low stress level		Moderate stress level		High stress level		
			n	%	N	%	n	%	
			31	16.4	150	79.4	8	4.2	
	n	%							0.198
Frequency of onychophagia:	Mild to Moderate								
	161	85.2	24	77.4	131	87.3	6	75.0	
	Severe								
	28	14.8	7	22.6	19	12.7	2	25.0	
Frequency of nail-biting episodes/day:	Mild to Moderate								1.000
	172	91.0	28	90.3	136	90.7	8	100.0	
	Severe								
	17	9.0	3	9.7	14	9.3	0	0.0	
Duration of nail-biting episodes/day:	Mild to Moderate								0.335
	175	92.6	27	87.1	140	93.3	8	100.0	
	Severe								
	14	7.4	4	12.9	10	6.7	0	0.0	

^*^Level of significance of Monte Carlo Exact Test

Table 6 represents logistic regression analysis of factors associated with nail-biting behaviour; all predictor variables were initially included in the logistic regression model to assess their influence on nail-biting behaviour (onychophagia). After removing the least significant variable (academic year), the final model included three predictors: smoking habit, family history of onychophagia, and fear of public spaces. The model was statistically significant (*p* < 0.001), indicating that these predictors collectively influenced the outcome. The Hosmer-Lemeshow test suggested an adequate fit (*p* = 0.948). The Nagelkerke R^2^ was 0.192, indicating that approximately 19.2% of the variability in nail-biting behaviour was explained by the model, while the remaining 80.8% could be attributed to other factors not included in the analysis.

**Table 6:** Logistic regression analysis of factors associated with nail-biting behaviour

Variables in the Equation	B	S.E	Wald	Sig	Exp(B)	95% C.I. for EXP(B)	
						Lower	Upper
Smoking habit (1)	1.567	0.654	5.748	0.017*	4.792	1.331	17.255
Family history of onychophagia (1)	1.574	0.336	21.929	0.000*	4.826	2.497	9.325
Fear of public spaces (1)	1.782	0.558	10.193	0.001*	5.943	1.990	17.750
Constant	- 0.225	0.135	2.757	0.097	0.799		

## Discussion

Despite the potential significance of nail biting among university students, little is known about its prevalence and associated factors among undergraduates in general and medical students in particular.

In the present study, a notably high prevalence of current onychophagia (57.3%) was observed among medical students. This finding aligns with a nearby study by Pacan et al. (2014), which reported a prevalence of 46.9% among medical students—comprising 19.2% active and 27.7% former nail biters [3]. Similarly, Hansen et al. (1990) documented that 63.6% of students in the United States reported nail biting at least twice a week [27]. Conversely, other studies have found significantly lower rates. For instance, Lesinskiene et al. (2021) noted that only 25% of physicians surveyed had a history of nail biting [22]. Erdogan et al. (2021) reported a prevalence of 27.8% among university students [23], while a study conducted in Ajman, United Arab Emirates, found that 20.7% of university students exhibited onychophagia [28]. The prevalence of onychophagia varies due to differences in study populations, methods, and cultural factors. Underreporting caused by embarrassment or lack of medical concern also contributes to this variability.

Among students who exhibited onychophagia, 63.5% were male and 36.5% were female; however, this difference was not statistically significant. These findings are in line with those of Shin et al. (2022), who reported a male predominance (73.6%) among individuals with onychophagia in a Korean cohort [29]. In contrast, Lesinskiene et al. (2021) observed a higher prevalence of nail biting among females, although the gender difference did not reach statistical significance [22]. The literature remains inconclusive on the influence of gender, with some studies indicating no significant association between gender and nail-biting behaviour [30–32], while others report a higher prevalence among females [33]. Variations in findings suggest that gender alone may not consistently predict onychophagia, and that more influential determinants may include cultural, psychological, or environmental factors.

In the present study, students who reported nail-biting behaviour were significantly more likely to have family members with the same habit compared to their non-nail-biting counterparts (*p* = 0.001). A familial history of onychophagia may point toward a potential genetic susceptibility, while observational learning, particularly through the replication of parental behaviours, is also regarded as a contributing etiological factor. Moreover, smoking was observed to be significantly more common among individuals with onychophagia (*p* = 0.006). This observation is consistent with findings by Erdoğan et al. (2021), who also reported a high prevalence of smoking among students with nail-biting behaviour [23]. Such associations may be explained by stress-related coping mechanisms or familial behavioural modelling, both of which may similarly influence the development of onychophagia.

Our study found a higher prevalence of onychophagia among students with a fear of public spaces, though no significant link was observed with broader anxiety disorders or specific fears. The absence of formal psychiatric assessments limits definitive conclusions regarding participants’ mental health. These results are consistent with Pacan et al. (2014), who reported no significant association between nail biting and clinical anxiety diagnoses [3]. Literature on this topic remains inconclusive, with only a minority of individuals with onychophagia meeting anxiety disorder criteria. Additionally, Ghanizadeh (2008) noted a substantial occurrence of psychiatric illnesses, particularly depression, among parents of children with nail-biting behaviour [15]. These findings suggest that while nail biting may be linked to certain fears, it is not consistently associated with formal anxiety disorders. Family mental health history was associated with nail-biting behaviour, which may reflect shared environmental or genetic influences.

Despite the well-documented association between onychophagia and elevated stress levels, the current study found no statistically significant difference in overall Perceived Stress Scale (PSS) scores between students who bite their nails and those who do not. Also, the level of stress, either mild/ moderate or severe, didn’t affect the onychophagia frequency or the duration of the episodes among nail biters. This finding stands in contrast to the results reported by Erdogan et al. (2021), who observed significantly higher median PSS scores among university students with onychophagia compared to non-biters [23]. One possible explanation for this discrepancy could be that nail biting, while associated with stress, may not always co-occur with high levels of perceived stress; in some individuals, it might function more as a habitual or compulsive behaviours rather than a direct response to psychological distress.

Interestingly, while the total PSS scores did not differ significantly between students with and without onychophagia, one specific item *“*Being angered because of things that were outside of his/her control*”* - showed a significant difference (*p* = 0.007). This suggests that although general stress levels were comparable across groups, students with onychophagia may experience particular difficulties in managing frustration or anger when faced with uncontrollable situations. Such emotional responses could act as situational triggers for nail-biting episodes rather than reflecting overall elevated stress. Therefore, the finding highlights that nail biting may be linked more to specific emotional regulation challenges than to global perceived stress.

## Conclusion and recommendations

Onychophagia is prevalent among medical students and is associated with family history, smoking, and fear of public spaces. While most students reported moderate stress levels, stress severity was not significantly linked to the frequency or duration of nail-biting. Behavioural interventions as habit-reversal training, along with educational initiatives and targeted support for high-risk students, may help reduce nail-biting. Integrating screening into wellness programs and providing access to counselling could be beneficial. Future longitudinal and interventional studies are needed to clarify causal relationships and assess effective strategies, while supportive academic environments may further mitigate compulsive behaviours.

## Limitation of the study

This study has several limitations. First, participants were recruited using non-randomized convenience sampling from a single university, which may introduce selection bias and limit the generalizability of the findings. Second, the Perceived Stress Scale (PSS) was modified by using only 8 of the 10 original items, which may affect the validity of the stress measurements and limit comparability with other studies using the full scale. Third, psychological conditions, including agoraphobia, and nail-related behaviours were self-reported, without clinical confirmation, which may affect the accuracy of these data. Fourth, the study did not specify the degree or side (maternal/paternal) of family history of onychophagia, limiting the assessment of familial patterns. Finally, because this study is cross-sectional, causal relationships cannot be determined. Another limitation of this study, is the academic achievement of the studied students assessed through the academic performance measures (e.g., GPA) was not collected in the study.

## Data Availability

Data is provided within the manuscript and within supplementary information files.

## References

[1] Lee DK, Lipner SR. Update on diagnosis and management of onychophagia and onychotillomania. Int J Environ Res Public Health. 2022;19(6):3392. 10.3390/ijerph19063392. PMID: 35329078; PMCID: PMC8953487. 35329078 10.3390/ijerph19063392 PMC8953487

[2] de Berker D. Childhood nail diseases. Dermatol Clin. 2006;24:355–63. 16798433 10.1016/j.det.2006.03.003

[3] Pacan P, Grzesiak M, Reich A, Kantorska-Janiec M, Szepietowski JC. Onychophagia and onychotillomania: prevalence, clinical picture and comorbidities. Acta Derm Venereol. 2014;94:67–71. 23756561 10.2340/00015555-1616

[4] Shetty SR, Munshi AK. Oral habits in children - a prevalence study. J Indian Soc Pedod Prev Dent. 1998;16:61–6. 11813757

[5] Williams Tl, Rose R, Chisholm S. What is the function of nail biting: an analog assessment study. Behav Res Ther. 2006;45:989–95. 17010305 10.1016/j.brat.2006.07.013

[6] Pacan P, Grzesiak M, Reich A, Szepietowski CJ. Onychophagia as a spectrum of obsessive-compulsive disorder. Acta Denn Venereol. 2009;89:278–80. 10.2340/00015555-0646 19479125

[7] Kolawole KA, Folayan MO, Agbaje HO, Oyedele TA, Onyejaka NK, Oziegbe EO. Oral habits and malocclusion in children resident in Ile-Ife Nigeria. Eur Arch Paediatr Dent. 2019;20:257–65. 30506282 10.1007/s40368-018-0391-3

[8] Rai A, Koirala B, Dali M, Shrestha S, Shrestha A, Niraula SR. Prevalence of oral habits and its association with malocclusion in primary dentition among school going children of Nepal. J Clin Pediatr Dent. 2022;46:44–50. 35311976 10.17796/1053-4625-46.1.8

[9] Aloumi A, Alqahtani A, Darwish A. Oral parafunctional habits among preschool children in Riyadh, Saudi Arabia. Saudi J Oral Sci. 2018;5:22.

[10] Halteh P, Scher RK, Lipner SR, Onychophagia. A nail-biting conundrum for physicians. J Dermatolog Treat. 2017;28(2):166–72. 27387832 10.1080/09546634.2016.1200711

[11] Sherif HD. Prevalence of different types of oral habits among school-children aged 6–12 years in Alexandria (a survey study). Egypt Orthodontic J. 2020;58:36–49.

[12] Ghanizadeh A, Shekoohi H. Prevalence of nail biting and its association with mental health in a community sample of children. BMC Res Notes. 2011;4:116. 21481256 10.1186/1756-0500-4-116 PMC3082216

[13] Ghanizadeh A. Nail biting; aetiology, consequences and management. Iran J Med Sci. 2011;36:73–9. 23358880 PMC3556753

[14] Penzel F. Skin picking and nail biting: related habits. Articles by Western Suffolk Psychological Service. [updated 11 May 2008]. Available from: http://westsuffolkpsych.homestead.com/SkinPicking.html

[15] Ghanizadeh A. Association of nail biting and psychiatric disorders in children and their parents in a psychiatrically referred sample of children. Child Adolesc Psychiatry Ment Health. 2008;2(1):13. 10.1186/1753-2000-2-13. 18513452 10.1186/1753-2000-2-13 PMC2435519

[16] O’Connor K, Lavoie M, Desaulniers B, Audet J-S. Cognitive Psychophysiological treatment of bodily-focused repetitive behaviours in adults: an open trial. J Clin Psychol. 2018;74:273–85. 28815684 10.1002/jclp.22501

[17] Grant JE, Mancebo MC, Eisen JL, Rasmussen SA. Impulse control disorders in children and adolescents with obsessive compulsive disorder. Psychiatry Res. 2010;30:109–13. 10.1016/j.psychres.2009.04.006 PMC2815218 20004481

[18] Nestadt G, Addington A, Samuels J. The identification of OCD-related subgroups based on comorbidity. Biol Psychiatry. 2003;53:914–20. 12742679 10.1016/s0006-3223(02)01677-3

[19] World Health Organization. The ICD-10: classification of mental and behavioural disorders: clinical description and diagnostic guidelines. Geneva: WHO; 1992.

[20] American Psychiatric Association. Diagnostic and statistical manual of mental disorders. (5th Edition), American Psychiatric Association, Arlington, VA. (2013).

[21] Baghchechi M, Pelletier JL, Jacob SE. Art of prevention: the importance of tackling the nail-biting habit. Int J Women’s Dermatol. 2020;7:309–13. 32964094 10.1016/j.ijwd.2020.09.008 PMC7497389

[22] Lesinskiene S, Pociute K, Dervinyte-Bongarzoni A, Kinciniene O. Onychophagia as a clinical symptom: A pilot study of physicians and literature review. Sci Prog. 2021;104(4):368504211050288. 10.1177/00368504211050288. PMID: 34874802; PMCID: PMC10373863. 34874802 10.1177/00368504211050288 PMC10373863

[23] Erdogan HK, Arslantas D, Atay E, Eyuboglu D, Unsal A, Dagtekin G, Kilinc A. Prevalence of onychophagia and its relation to stress and quality of life. Acta Dermatovenerol Alp Pannonica Adriat. 2021;30(1):15–9. PMID: 33765752. 33765752

[24] Reis RS, Hino AA, Añez CR. Perceived stress scale: reliability and validity study in Brazil. J Health Psychol. 2010;15(1):107–14. 10.1177/1359105309346343. 20064889 10.1177/1359105309346343

[25] Mitchell AM, Crane PA, Kim Y. Perceived stress in survivors of suicide: psychometric properties of the perceived stress scale. Res Nurs Health. 2008;31(6):576–85. 10.1002/nur.20284. 18449942 10.1002/nur.20284

[26] Barbosa-Leiker C, Kostick M, Lei M, McPherson S, Roper V, Hoekstra T, Wright B. Measurement invariance of the perceived stress scale and latent mean differences across gender and time. Stress Health. 2013;29(3):253–60. 10.1002/smi.2463. 23027679 10.1002/smi.2463

[27] Hansen DJ, Tishelman AC, Hawkins RP, Doepke KJ. Habits with potential as disorders. Behav Modif. 1990;14(1):66–80. 10.1177/01454455900141005. 2294902 10.1177/01454455900141005

[28] Elattar H, Elhiny O, El Janah M. Different types of malocclusion and oral habits in Sharja and Ajman Emirates. Egypt Orthodontic J. 2019;55(6):39–52. 10.21608/eos.2019.77126.

[29] Shin JO, Roh D, Son JH, Shin K, Kim HS, Ko HC, Kim BS, Kim MB. Onychophagia: detailed clinical characteristics. Int J Dermatol. 2022;61(3):331–6. 10.1111/ijd.15861. Epub 2021 Aug 20. PMID: 34416026. 34416026 10.1111/ijd.15861

[30] Winebrake JP, Grover K, Halteh P, Lipner SR. Paediatric onychophagia: a survey-based study of prevalence, aetiologies, and co-morbidities. Am J Clin Dermatol. 2018;19:887–91. 30171499 10.1007/s40257-018-0386-1

[31] Gur K, Erol S, Incir N. The effectiveness of a nail-biting prevention program among primary school students. J Spec Pediatr Nurs. 2018;23:e12219. 29797491 10.1111/jspn.12219

[32] Almeida IA, Jeske S, Mesemburg MA, Berne MEA, Villela MM. Prevalence of and risk factors for intestinal parasite infections in paediatric patients admitted to public hospitals in Southern Brazil. Rev Soc Bras Med Trop. 2017;50:853–6. 29340467 10.1590/0037-8682-0116-2017

[33] Malone AJ, Massler M. Index of nail-biting in children. J Abnorm Soc Psychol. 1982;47:192–202.

